# A Census Tract–Level Examination of Social Determinants of Health among Black/African American Men with Diagnosed HIV Infection, 2005–2009—17 US Areas

**DOI:** 10.1371/journal.pone.0107701

**Published:** 2014-09-30

**Authors:** Zanetta Gant, Larry Gant, Ruiguang Song, Leigh Willis, Anna Satcher Johnson

**Affiliations:** 1 Division of HIV/AIDS Prevention, Centers for Disease Control and Prevention, National Center for HIV/AIDS, Viral Hepatitis, STD, and TB Prevention, Atlanta, Georgia, United States of America; 2 School of Social Work, University of Michigan, Ann Arbor, Michigan, United States of America; UCSF, United States of America

## Abstract

**Background:**

HIV disproportionately affects black men in the United States: most diagnoses are for black gay, bisexual, and other men who have sex with men (collectively referred to as MSM). A better understanding of the social conditions in which black men live and work may better explain why HIV incidence and diagnosis rates are higher than expected in this population.

**Methods:**

Using data from the National HIV Surveillance System and the US Census Bureau's American Community Survey, we examined the relationships of HIV diagnosis rates and 5 census tract–level social determinants of health variables for 21,948 black MSM and non-MSM aged ≥15 years residing in 17 areas in the United States. We examined federal poverty status, marital status, education level, employment status, and vacancy status and computed rate ratios (RRs) and prevalence odds ratios (PORs), using logistic regression with zero-inflated negative binomial modeling.

**Results:**

Among black MSM, HIV diagnosis rates decreased as poverty increased (RR: 0.54). At the time of HIV diagnosis, black MSM were less likely than black non-MSM to live in census tracts with a higher proportion below the poverty level (POR: 0.81) and with a higher proportion of vacant houses (POR: 0.86). In comparison, housing vacancy was positively associated with HIV diagnosis rates among black non-MSM (RR: 1.65). HIV diagnosis rates were higher for black MSM (RR: 2.75) and non-MSM (RR: 4.90) whose educational level was low. Rates were significantly lower for black MSM (RR: 0.06) and non-MSM (RR: 0.26) as the proportion unemployed and the proportion married increased.

**Conclusions:**

This exploratory study found differences in the patterns of HIV diagnosis rates for black MSM and non-MSM and provides insight into the transmission of HIV infection in areas that reflect substantial disadvantage in education, housing, employment, and income.

## Background

HIV disproportionately affects black/African American (black) men who accounted for 12% of the male population and 42% of HIV infections diagnosed among men in the United States during 2011 [1], [2]. In 2011, estimated rates of diagnoses of HIV infection among black men were nearly 8 times the rate for whites and more than twice the rate for Hispanics/Latinos. For black men, the lifetime risk of HIV is estimated to be 1 in 16 [3]. Gay, bisexual, and other men who have sex with men (collectively referred to as MSM) account for the highest number of cases among black men [1], [2]. Although the rates of HIV infection are higher for black MSM than for MSM of any other race/ethnicity, research has shown no significant differences in the sexual risk behaviors of black and white MSM [4]–[6]. This observation suggests that factors other than individual risk behavior are contributing to the rate disparity. These disproportionate rates of HIV may be partly explained by a better understanding of the social determinants that affect black men's lives and work [7]–[11]. Social determinants of health (SDH) serve as accessible proxies for social factors (e.g., poverty, education, marital status, and employment) and physical environmental factors (e.g., property values, housing vacancies), and they allow us to contextualize HIV risk among at-risk populations [12]–[15].

Research addressing disproportionate rates of HIV infection among black men has focused mainly on the sexual behaviors of black MSM [6], [16], [17], not on the environment in which these behaviors occur. This research tends to assess the risk of HIV infection solely by examining sexual behavior such as unprotected anal intercourse. Whereas, most studies examining the risk of HIV infection among heterosexual black men have not assessed risk solely with a specific sexual behavior [8], [10], [18], [19]; instead they compound behavioral risk factors with other factors such as drug use, crime, and excess mortality [8], [11], [20], [21]. This assessment may not truly encompass the behavioral and environmental paradigms that exist for heterosexual black men. Additionally, most previous research that has examined HIV risk among the black male population has addressed MSM only or included MSM and non-MSM as a combined population. Given that black MSM accounted for approximately 70% of newly diagnosed HIV infections among black males annually [1], [22], examination of the combined populations could skew the conclusions toward those applicable to the MSM population.

We had 2 objectives: (1) to assess selected SDH that may provide insight into the disparate rates of HIV transmission among black MSM and non-MSM subpopulations separately; and (2) to examine the differences in proportion of HIV diagnoses between black MSM and non-MSM and the SDH that may be associated with these differences.

## Methods

Data were obtained from 3 sources: the Centers for Disease Control and Prevention's (CDC's) National HIV Surveillance System (NHSS) (http://www.cdc.gov/nchhstp/Atlas/Index.htm), CDC's HIV Geographic Information System (GIS) Supplemental Surveillance Project (http://www.cdc.gov/hiv/pdf/statistics_2005_2009_HIV_Surveillance_Report_vol_18_n4.pdf), and the US Census Bureau's American Community Survey (ACS) (http://www.census.gov/acs/www/data_documentation/data_main/). The numbers, percentages, and rates of diagnoses of HIV infection are based on cases reported to NHSS through December 2010 and included cases among black males aged 15 years and older whose HIV infection was diagnosed during 2005–2009, regardless of the stage of disease at diagnosis. The population of black MSM included men who had ever had sexual contact with other men, men who had ever had sexual contact with both men and women, and men who had sexual contact with other men and injected drugs. The population of black non-MSM included men with infection attributable to heterosexual contact (i.e., men who had ever had heterosexual contact with a person known to have, or to be at high risk for, HIV infection), injection drug use (IDU), or other modes of transmission (i.e., hemophilia, blood transfusion, and perinatal exposure).

The analysis was based on data from the 17 (of 29) areas that were funded for the 2010 HIV GIS Supplemental Surveillance Project and that provided 5 years (2005–2009) of geocoded data for residence at diagnosis: Colorado, District of Columbia, Illinois (excluding Chicago), Iowa, Los Angeles County, Louisiana, Michigan, Minnesota, Mississippi, New York City, New York State, North Carolina, San Francisco, South Carolina, Virginia, Washington, and Wisconsin. We linked census tract SDH indicator data to individual cases on the basis of residence at the time of diagnosis. HIV diagnosis data were statistically adjusted for missing transmission category [23], but not for delayed or incomplete reporting.

Census tract–level SDH data were obtained from the ACS 2005–2009 5-year estimates [24]. We examined 5 SDH variables: federal poverty status, marital status, education level, employment status, and housing vacancy status. We measured

- federal poverty status by the “proportion of residents in the census tract who were living below the US poverty level (i.e., below a specified threshold) within the last 12 months of the survey response”
- marital status by the “proportion of people currently married among individuals 15 years and older”
- education level by the “proportion with less than high school education for individuals 18 years and older”
- employment status by the “proportion in the workforce without a job for individuals 16 years and older”
- housing vacancy status by the “proportion of vacant houses within a census tract”

We included these SDH variables because they are generally recognized in the scientific literature as population determinants of health. Although other social determinants may affect health, research on social determinants among black males has emphasized the need to incorporate these specific structural and societal factors into analyses of public health data [15], [16], [25]. Additionally, authors of studies evaluating SDH have noted the need to include analysis of the geographic environment to help explain how these factors moderate individual-level behaviors that influence health outcomes [9], [26], [27].

Rates per 100,000 population were calculated for the numbers of diagnoses of HIV infection in each census tract. The population denominators used to compute these rates for the 17 areas were based on the US Census ACS 5-year estimates of the black male populations [24]. The 5-year average annual HIV diagnosis rate was calculated by dividing the total number of diagnoses among black males aged 15 years and older during 2005–2009 by the total black male population aged 15 years and older for the same 5 years. The result was then multiplied by 100,000 and divided by 5 to obtain the annualized 5-year rate. Because of the lack of US Census data stratified by transmission category, HIV diagnosis rates among black MSM and non-MSM were calculated by using the general black male population as the denominator.

The association between HIV diagnosis rates among black MSM and non-MSM and SDH variables was determined by using stratified logistic regression with zero-inflated negative binomial modeling [28]. Zero-inflated modeling was used to account for the excess of zero counts (i.e., census tracts with no black males with diagnosed HIV infection) to reduce bias due to the skewing of data toward zero counts. Rate ratios (RRs) were computed to determine the HIV diagnosis rates for black MSM and non-MSM when the proportion change for the SDH variable of interest (e.g., proportion of people living below poverty level) increased from zero (i.e., 0%, or no one living below poverty level) to one (i.e., 100%, or everyone living below poverty level). Consequently, we constructed 2 models and calculated average annual HIV diagnosis RRs and 95% confidence intervals (CIs) stratified for black MSM and non-MSM at the census tract–level. We report the effect of each SDH variable on the HIV diagnosis rate when the SDH variable was increased by 1 percentage point (i.e., 0.01).

Because we were unable to determine the total populations of black MSM and non-MSM in the 17 areas to compute transmission category–specific rates, we computed prevalence odds ratios (PORs). The POR was defined as the prevalence odds of HIV diagnosis for black MSM vs. non-MSM based on the census tract at the time of diagnosis, when the proportion change for the specified SDH variable of interest increased from 0 (or 0%) to 1 (or 100%). We calculated PORs and 95% CIs for HIV diagnosis among black non-MSM vs MSM, and we report the effect of each SDH variable on the prevalence odds of HIV diagnosis for black MSM vs non-MSM when the SDH variable increased by 1 percentage point (i.e., 0.01). The prevalence odds of HIV diagnosis for black MSM vs. non-MSM was defined as (No. MSM + 1)/(No. non-MSM + 1). The numeral 1 was added to the numerator and the denominator to avoid an undefined POR when the number of non-MSM was zero. A POR of>1 indicated that the proportion of black MSM compared with black non-MSM with diagnosed HIV increased as the proportion of the SDH variable of interest increased. However, a POR of <1 indicated that the proportion of black MSM compared with black non-MSM with diagnosed HIV decreased as the proportion of the SDH variable of interest increased.

Recognizing the complex relationships between these SDH variables, we retained all SDH variables in our models (i.e., we analyzed each individual social determinant while controlling for all other SDH) to determine the overall impact of contributing factors. We also controlled for 3 indicators with possible confounding effects in our model: proportion of males in the general population in a census tract, proportion of blacks in the general population in a census tract, and proportion of people aged 15–49 years in a census tract. All results for each outcome of interest in the logistic regression models are based on controlling for all other variables. Statistical analyses were based on regression with a significance level of 0.05 and were performed by using SAS version 9.2 (SAS Institute, Inc., Cary, NC). All reported outcomes were statistically significant unless otherwise noted.

## Results

During 2005–2009, in the 17 HIV Supplemental Surveillance Project areas, HIV infection was diagnosed for 22,396 black males aged 15 years and older, and residential addresses had been geocoded to the census tract level in 18,330 census tracts for 98% (21,948) (Table 1). Of the 15,276 (69.6%) classified as MSM, 29.4% were aged 18–24 years at diagnosis, and 28.3% were aged 25–34 years. Of the 6,672 black males classified as non-MSM, 31.6% were aged 45–54 years, and 29.0% were aged 35–44 years. The average annual HIV diagnosis rate for black males was 416.6 per 100,000 population.

**Table 1:** Diagnoses of HIV infection among black/African American MSM and non-MSM, by age at diagnosis, 2005–2009—17 areas.

	MSM		Non-MSM		Total	
	No.	%	No.	%	No.	Average annual rate^a^
Age at diagnosis, years						
15–17	380	2.5	33	0.5	413	103.5
18–24	4,484	29.4	383	5.7	4,866	564.2
25–34	4,321	28.3	1,029	15.4	5,349	542.1
35–44	3,459	22.6	1,936	29.0	5,394	551.9
45–54	1,965	12.9	2,110	31.6	4,075	435.0
55–64	552	3.6	900	13.5	1,452	242.2
≥65	116	0.8	282	4.2	398	78.6
**Total**	**15,276**		**6,672**		**21,948**	**416.6**

Note. Data include persons with diagnosed HIV infection regardless of stage of disease at diagnosis. HIV diagnosis data were statistically adjusted for missing transmission category, but not for reporting delays or incomplete reporting.

MSM, men who reported ever having had sexual contact with other men.

a Rates are per 100,000 population.

In the stratified logistic regression models, some of the patterns for the 2 populations of black males (Table 2) differed. For every percentage point increase in the proportion below the poverty level, the likelihood of HIV diagnosis among black MSM decreased by 0.46% [(RR_MSM_ =  0.54)–1]. The reverse pattern, although not statistically significant, was observed among non-MSM. For every percentage point increase in the proportion of vacant houses, the likelihood of HIV diagnosis among black non-MSM increased by 0.65% [(RR_non-MSM_ =  1·65)–1]. The reverse pattern, although not statistically significant, was observed among black MSM.

**Table 2:** HIV diagnosis rate ratios among black/African American MSM and non-MSM, by selected census tract–level social determinants of health, 2005–2009—17 areas.

	MSM			Non-MSM		
Proportion	Rate ratio	(95% CI)	P value	Rate ratio	(95% CI)	P value
General population of males	0.02	(0.01–0.04)	<0.0001	0.02	(0.01–0.03)	<0.0001
General population of blacks	0.78	(0.70–0.86)	<0.0001	1.17	(1.01–1.36)	0.04
Persons aged 15–49	4.98	(3.56–6.97)	<0.0001	2.51	(1.57–4.04)	0.0001
Below federal poverty level	0.54	(0.42–0.70)	<0.0001	1.17	(0.82–1.66)	0.38
Married	0.06	(0.04–0.08)	<0.0001	0.10	(0.07–0.15)	<0.0001
Less than high school education	2.75	(2.14–3.53)	<0.0001	4.90	(3.43–7.01)	<0.0001
Unemployed	0.26	(0.17–0.41)	<0.0001	0.06	(0.03–0.11)	<0.0001
Vacant houses	0.98	(0.72–1.32)	0.87	1.65	(1.09–2.49)	0.02

Note. Data include persons with diagnosed HIV infection regardless of stage of disease at diagnosis. HIV diagnosis data were statistically adjusted for missing transmission category, but not for reporting delays or incomplete reporting. All results for each outcome of interest in the models are based on controlling for all other variables.

MSM, men who reported ever having had sexual contact with other men.

CI, confidence interval.

In contrast, the stratified models revealed some similarities among black MSM and non-MSM (Table 2). For every percentage point increase in the proportion married and the proportion unemployed, the likelihood of HIV diagnosis among black MSM decreased by 0.94% [(RR_MSM_ = 0.06)–1] and 0.74% [(RR_MSM_ = 0.26)–1], respectively, and among black non-MSM, decreased by 0.90% [(RR_ non-MSM_ = 0·10)–1] and 0.94% [RR_non-MSM_ = 0·06)–1], respectively. For every percentage point increase in the proportion with less than a high school education, the likelihood of HIV diagnosis increased 1.75% [(RR_MSM_ = 2.75)–1] among black MSM and 3.90% [(RR_non-MSM_ = 4.90)–1] among black non-MSM.

From our POR calculations (Table 3), for every percentage point increase in the proportion below the poverty level, the proportion married, and the proportion of vacant houses, the change in odds of HIV diagnosis for black MSM versus non-MSM were lower by 0.19% (POR: 0.81), 0.16% (POR: 0.84), and 0.14% (POR: 0.86), respectively. However, for every percentage point increase in the proportion with less than a high school education, the odds of HIV diagnosis for black MSM versus black non-MSM were higher by 0·14% (POR: 1·14).

**Table 3:** Prevalence odds ratios^a^ of HIV infection diagnosis for black/African American MSM vs. non-MSM, by selected census tract-level social determinants of health (SDH), 2005–2009—17 areas.

Proportion	Prevalence odds ratio (POR)^b^	95% CI	P value
Below federal poverty	0.81	(0.75–0.87)	<0.0001
Married	0.84	(0.78–0.90)	<0.0001
Less than high school education	1.14	(1.06–1.22)	0.0002
Unemployed	0.90	(0.78–1.03)	0.11
Vacant houses	0.86	(0.80–0.93)	<0.0001

Note. Data include persons with diagnosed HIV infection regardless of stage of disease at diagnosis. HIV diagnosis data were statistically adjusted for missing transmission category, but not for reporting delays or incomplete reporting. All results for each outcome of interest in the models are based on controlling for all other variables.

MSM, men who reported ever having had sexual contact with other men.

CI, confidence interval.

a Black non-MSM is the reference group.

b The prevalence odds is defined as (#MSM+1)/(#non-MSM+1), where adding 1 to both the numerator and the denominator avoids the prevalence odds undefined when there are no diagnosed HIV infections among black non-MSM. PORs>1 indicates that among black males, as the proportion of a SDH variable of interest increases, the probability of black MSM diagnosed with HIV is higher compared to black non-MSM.

## Discussion

Our analysis is one of few large-scale studies examining SDH among black males on the basis of HIV transmission category. Overall, we found that HIV diagnosis rates followed differing patterns for black MSM and non-MSM when we examined poverty and housing vacancies. Similar patterns emerged when we examined education level, unemployment status, and marital status.

According to an earlier study of more than 9,000 people in 23 US cities, heterosexuals living below the poverty level were 5 times as likely as the nation's general population to be HIV-positive, regardless of race or ethnicity [29]. We found a similar, but statistically nonsignificant, pattern among black non-MSM in our analysis. Also, our results showed that as poverty increased, HIV diagnosis rates decreased among black MSM. Although previous research suggests that community involvement in HIV-related organizations and groups (e.g., social services, support groups) may protect against the association of poverty and risky sexual behavior [30], a more plausible explanation could be that poverty is masking HIV infection. Additionally, we found that at the time of HIV diagnosis, black MSM were less likely than black non-MSM to live in census tracts in which a higher proportion of persons were living below the poverty level. On the basis of geographic location, the structural barriers of poverty appear to have differing effects on HIV diagnosis rates among black MSM compared with non-MSM.

Housing vacancy, one of many measures of neighborhood distress, can serve as a proxy measurement of material deprivation, lack of resources, households in poverty, and socioeconomic status [31]–[33]. A geospatial methods study found that housing vacancy rates were positively associated with HIV diagnosis rates and high-risk heterosexual transmission rates [33]. Our study supports these findings for black non-MSM, but not for black MSM. Additionally, we found that at the time of diagnosis, black MSM were less likely than black non-MSM to live in census tracts with a higher proportion of vacant houses. Further assessment is needed to better understand the possible relationship between housing vacancies and HIV diagnosis rates among black MSM.

A low level of education (<high school education) followed the expected associations for black MSM and non-MSM: HIV diagnoses rates for black men were higher when educational attainment was low [34]–[38]. Despite the similar pattern for both populations and contrary to our expectations, our results show that at the time of HIV diagnosis, MSM were more likely than non-MSM to live in census tracts in which residents had a lower level of education. This finding is consistent with the findings of other studies in which patterns of lower educational access, performance, and outcomes persisted for black MSM compared with black non-MSM [39], [40]. Persistent fears and documented reports of harassment and physical violence toward gay and bisexual youth in educational settings leads to increased missed days of school and dropouts at all educational levels (i.e., presecondary, secondary, and postsecondary) [39], [40]. Additionally, a relationship may exist between low educational attainment and the proportion of persons who live below the poverty level. Further research might examine the extent to which the interaction of these structural factors affects rates of HIV infection among black MSM and non-MSM.

Unemployment rates and HIV infection varied in ways that we did not expect. We expected unemployment to follow the same pattern as education for black MSM and non-MSM, but HIV diagnosis rates were significantly lower as unemployment rates increased. We suspect that unemployed persons may not be able to access HIV testing and treatment and therefore do not get tested. For example, Mayer et al. found that unemployed black MSM were more likely to have undiagnosed HIV infection [25]. If rates of risky behaviors are similar among employed and unemployed MSM, then lower rates of testing among unemployed MSM could explain lower rates of HIV diagnosis in that group. Employed persons have a greater chance of receiving testing through their jobs and are more likely to be engaged in the health care system through the provision of employer-sponsored health insurance.

Furthermore, a relationship may exist between the proportion unemployed and the proportion living below the poverty level. This relationship could suggest that impoverished men have lower social mobility and increased chances for unemployment, thus fewer opportunities to meet prospective partners. Regardless of the outcome of partner engagement (e.g. short-term or long-term relationships), possession of, or access to, material resources and economic opportunities may be a critical component in partner seeking and partner engagement. As previously discussed, poverty may be masking HIV infections among black MSM. Additional research is needed to further assess the relationship between unemployment and HIV diagnosis rates.

For black MSM and non-MSM, HIV diagnosis rates decreased as the proportion of married persons in the census tract increased. At the time of diagnosis, black MSM were less likely than non-MSM to live in a census tract in which more persons were married. The protective effects of marriage (e.g., reduction in the number of partners, sex with partner at low risk) for heterosexual partners continues to be debated [41], [42] but appears to be supported by our findings. It is unclear how marriage would protect black MSM, given the absence of legalized marriage among same-sex partners in some of the participating states during the time frame of data collection. It is possible that the rates of HIV diagnosis among MSM were low because the populations of MSM who lived in the census tracts in these 17 areas were small; however, this question could not be answered because we were unable to compute transmission category–specific HIV rates by census tract. Little explanation in current relevant literature could be found regarding the latter finding, and should be a focus of further study and possibly an in-depth examination of some areas to assess the relationship.

Our analysis had several limitations. First, diagnoses of HIV infection do not represent incidence, or new infections. The time from infection to diagnosis varies by individual, and residence at HIV diagnosis may not be the residence at the time HIV infection was acquired. Second, data for this analysis were not adjusted for reporting delays. This may have resulted in an underestimate of the number of cases diagnosed during 2005–2009. Third, data were limited to persons in 17 areas whose residential addresses were complete and thus could be geocoded; therefore, results may not reflect the population of black males with diagnosed HIV in those areas. However, according to the surveillance databases in these 17 areas, 98% of persons with diagnosed HIV had residential addresses that were geocoded. Fourth, the US Census Bureau does not collect data by transmission category (e.g., injection drug use) or sexual orientation (e.g., MSM). Our estimated HIV rates for black MSM and non-MSM are based on a denominator population of all black males aged 15 years and older. Although a previous study estimated that approximately 2.0% of the US population are MSM [43], we could not extrapolate the percentage of MSM in the 17 areas or in the US black male population. Finally, given that SDH information is not available at the level of the individual, we used census tract data as a surrogate for the environment in which persons with diagnosed HIV infection live.

## Conclusions

This exploratory study shows the importance of understanding how SDH may frame HIV diagnosis rates among black men. These determinants may provide greater knowledge about where, when, and how to tailor and deploy prevention and care resources to populations at risk, particularly black males. Although, some of the patterns of HIV rates and SDH among black MSM and non-MSM in this study could not be easily explained, they highlight the current gaps in SDH research for this population, thereby identifying the need for additional research to better understand how SDH affects HIV risk.

Overall, we found differences in the patterns of HIV diagnosis rates for black MSM and non-MSM. These differences provide insight into the transmission of HIV infection in areas that reflect substantial disadvantage in education, housing, employment, and income. Differing patterns among these SDH also show the importance of examining several determinants together to better understand their associations with risk behavior in the population of interest. Although causality could not be determined in this study, this study opens up opportunity for further research to address structural differences that may affect HIV infection among black MSM and non-MSM.
